# Perspective: An Extension of the STROBE Statement for Observational Studies in Nutritional Epidemiology (STROBE-nut): Explanation and Elaboration

**DOI:** 10.3945/an.117.015941

**Published:** 2017-09-07

**Authors:** Agneta Hörnell, Christina Berg, Elisabet Forsum, Christel Larsson, Emily Sonestedt, Agneta Åkesson, Carl Lachat, Dana Hawwash, Patrick Kolsteren, Graham Byrnes, Willem De Keyzer, John Van Camp, Janet E Cade, Darren C Greenwood, Nadia Slimani, Myriam Cevallos, Matthias Egger, Inge Huybrechts, Elisabet Wirfält

**Affiliations:** 1 Department of Food and Nutrition, Umeå University, Umeå, Sweden;; 2 Department of Food and Nutrition, and Sport Science, University of Gothenburg, Gothenburg, Sweden;; 3 Department of Clinical and Experimental Medicine, Linköping University, Linköping, Sweden;; 4 Department of Clinical Sciences in Malmö, Lund University, Malmö, Sweden;; 5 Institute of Environmental Medicine, Karolinska Institutet, Stockholm, Sweden;; 6 Department of Food Safety and Food Quality, Ghent University, Ghent, Belgium;; 7 International Agency for Research on Cancer, Lyon, France;; 8 Department of Biosciences and Food Sciences, University College Ghent, Ghent, Belgium;; 9 Nutritional Epidemiology Group, School of Food Science and Nutrition, and; 10 Biostatistics Unit, School of Medicine, University of Leeds, Leeds, United Kingdom; and; 11 Department of Clinical Research and; 12 Institute of Social and Preventive Medicine, University of Bern, Bern, Switzerland

**Keywords:** dietary assessment, checklist, epidemiology, nutrition, reference standards, scientific reporting

## Abstract

Nutritional epidemiology is an inherently complex and multifaceted research area. Dietary intake is a complex exposure and is challenging to describe and assess, and links between diet, health, and disease are difficult to ascertain. Consequently, adequate reporting is necessary to facilitate comprehension, interpretation, and generalizability of results and conclusions. The STrengthening the Reporting of OBservational studies in Epidemiology (STROBE) statement is an international and collaborative initiative aiming to enhance the quality of reporting of observational studies. We previously presented a checklist of 24 reporting recommendations for the field of nutritional epidemiology, called “the STROBE-nut.” The STROBE-nut is an extension of the general STROBE statement, intended to complement the STROBE recommendations to improve and standardize the reporting in nutritional epidemiology. The aim of the present article is to explain the rationale for, and elaborate on, the STROBE-nut recommendations to enhance the clarity and to facilitate the understanding of the guidelines. Examples from the published literature are used as illustrations, and references are provided for further reading.

## Introduction

The need for specific reporting recommendations for dietary studies has been highlighted (1, 2), because both the exposure in itself (i.e., the habitual dietary intake) and its assessment are complex and multifaceted. Poor reporting in nutritional epidemiology could result in the failure to replicate studies, cause readers to draw erroneous conclusions from research findings, and potentially result in misleading interpretation of how diet affects human health, with the risk of inferring incorrect public health messages. Clear research reports will facilitate correct interpretation of study findings and provide essential information enabling full consideration of research findings in meta-analyses.

Essential elements of the reporting are clear descriptions of the study design (**Text Box 1**), the specific dietary assessment methodology, and the measures taken during data collection and handling, as well as during statistical analysis. The accuracy and biases of self-reported dietary intakes are largely consequences of the dietary assessment methodology and its format, the increasing variety of foods available, and the willingness and ability of the respondent to accurately report food intake. These characteristics of dietary data, together with the naturally very large within-person variation in dietary intakes in most populations, require attention.

**TEXT BOX 1 STUDY DESIGN FOR NUTRITIONAL EPIDEMIOLOGY**

The general principles of epidemiology (3) also apply to nutritional epidemiology, a subdiscipline of epidemiology seeking to understand the role of diet and nutrition in relation to health outcomes. The 3 major designs in observational nutritional epidemiology are the cohort, case-control, and cross-sectional designs. The aims of studies that use these designs are either to evaluate the association between dietary exposures and disease risk (i.e., etiologic and analytical epidemiology) or to describe the dietary intakes and nutritional status in a population. Because the currently available dietary assessment methods have different characteristics and utility (see Nut-8.1 and Text Box 2), the study aim and design will have major implications for the choice of dietary assessment methodology. Observational studies that wish to examine if population groups are at nutritional risk, or if dietary deficiencies are present, may benefit from considering the classical “ABCD rule of thumb” for nutritional assessment that includes measures of anthropometry, biochemistry, and clinical signs, in addition to those of dietary intake data (4).

In a cohort study, participants are followed over time. Dietary exposures are assessed at baseline and may be assessed repeatedly during follow-up, and the occurrence of outcomes is ascertained during follow-up. Subjects with various degrees of exposure are compared (e.g., high exposure compared with low exposure) for the estimations of risk and rate of disease or disease-related outcomes.

In a case-control study, persons with and without a particular disease are studied and the odds of the dietary exposure are compared among the cases and controls to obtain the OR. The OR is interpreted as the risk ratio, rate ratio, or prevalence OR, depending on the sampling strategy and the nature of the population studied. In traditional case-control designs, the exposure is assessed retrospectively with respect to the time of disease initiation. This is an important limitation, because one cannot be sure that the dietary exposure preceded the outcome, and the reported dietary intakes among cases may be influenced by knowledge about the disease (i.e., recall bias; see Text Box 5). In contrast, case-control studies nested within large cohort studies have the advantage of using the data collected during the baseline examinations of the cohort study, thus avoiding the disadvantages of retrospective data collection.

Both cohort studies and case-control studies evaluate the link between diet and disease, and both study designs therefore require dietary data that make it possible to rank-order individuals on their estimated usual intakes. This means that FFQs, dietary histories, repeated 24-h recalls, or repeated food records (diaries) are the dietary assessment methods of choice (see Text Box 2).

Cross-sectional studies are useful for descriptive purposes that aim to present the prevalence of exposures and health conditions. However, an observed association may be misleading because the temporal relation between exposure and outcome cannot be determined, and also because persons with less severe disease of long duration accumulate, whereas those with aggressive disease are likely to die early. Cross-sectional studies are suitable to describe the dietary intake distribution in a population, to evaluate the proportion of a population at risk of inadequate intakes or intakes below or above the recommendation, and also for validation purposes. These study aims require absolute intake data to estimate mean intakes for individuals and groups, and repeated food records or repeated 24-h recalls (see Text Box 2) are therefore the most suitable dietary assessment methods. Cross-sectional study designs are also used when dietary data are evaluated in relation to biomarkers of exposure, or disease intermediates, but such projects only need to rank-order individuals on usual intakes, not estimate mean intakes.

Ecological studies describe the relation between diet and health outcomes on a highly aggregated level and do not consider intakes of the individual. Instead, readily available information is used, such as food balance sheets (5), food disappearance data (e.g., average per capita food or nutrient intakes across countries), or household budget surveys. These food data are examined together with national health statistics. Such studies can therefore solely generate hypotheses and will not provide any meaningful estimates of diet-disease causal associations. The information can also be expressed as trends over time within a country, region, or household. The danger with this type of study is ecological fallacy, in which inferences about individuals are deduced from inferences about the group when, in reality, the 2 variables of interest may not be related at all.

## The STROBE Statement and the STROBE-nut Extension

The need for high-quality reporting of research findings led to important initiatives, such as the STrengthening the Reporting of OBservational studies in Epidemiology (STROBE) (6). The STROBE statement is the outcome of an international collaboration established in 2004, which resulted in a set of 22 evidence-based recommendations for reporting of observational studies, and is currently endorsed by >100 journals. An accompanying elaboration and explanation article was also published (3). Like all reporting guidelines, the STROBE recommendations are neither prescriptions for the design or conduct of studies nor a set of guidelines to evaluate the quality of observational research. Rather, STROBE ought to be seen as recommendations to enhance the quality, completeness, and transparency of the reporting of observational studies. Several extensions of the STROBE statement have been developed [e.g., STROBE for molecular epidemiology studies (STROBE-ME); see http://strobe-statement.org (7) for a complete list].

The STROBE-nut (1, 2) is a nutritional epidemiology extension of the original STROBE statement. Its development was coordinated by a multidisciplinary group of 21 experts through a systematic process, including 3 Delphi rounds with external experts. The STROBE-nut includes a checklist that comprises 24 recommendations (**Table 1**) with the intention to improve the reporting quality and completeness of observational studies with regard to diet and health. A table to aid reporting is available on the STROBE-nut website [http://www.strobe-nut.org (8)] and added as a **Supplemental Reporting Table** to this article.

**TABLE 1:** STROBE-nut: an extension of the STROBE statement for nutritional epidemiology^1^

Item	Item number	STROBE recommendations	STROBE-nut
Title and abstract	1	(a) Indicate the study’s design with a commonly used term in the title or the abstract. (b) Provide in the abstract an informative and balanced summary of what was done and what was found.	Nut-1. State the dietary/nutritional assessment method(s) used in the title or in the abstract.
Introduction			
Background rationale	2	Explain the scientific background and rationale for the investigation being reported.	—
Objectives	3	State specific objectives, including any prespecified hypotheses.	—
Methods			
Study design	4	Present key elements of the study design early in the paper.	—
Settings	5	Describe the setting, locations, and relevant dates, including periods of recruitment, exposure, follow-up, and data collection.	Nut-5. Describe any characteristics of study settings that might affect the dietary intake or nutritional status of the participants, if applicable.
Participants	6	(a) Cohort study: Give the eligibility criteria and the sources and methods of selection of participants. Describe methods of follow-up. Case-control study: Give the eligibility criteria and the sources and methods of case ascertainment and control selection. Give the rationale for the choice of cases and controls. Cross-sectional study: Give the eligibility criteria, and the sources and methods of selection of participants. (b) Cohort study: For matched studies, give matching criteria and number of exposed and unexposed. Case-control study: For matched studies, give matching criteria and the number of controls per case.	Nut-6. Report any particular dietary, physiologic, or nutritional characteristics considered when selecting the target population.
Variables	7	Clearly define all outcomes, exposures, predictors, potential confounders, and effect modifiers. Give diagnostic criteria, if applicable.	Nut-7.1. Clearly define foods, food groups, nutrients, or other food components (e.g., preparation method, taxonomical descriptors, classification, chemical form).
			Nut-7.2. When calculating dietary patterns, describe the methods to obtain them and their nutritional properties.
Data sources and measurements	8	For each variable of interest, give sources of data and details of methods of assessment (measurement).	Nut-8.1. Describe the dietary assessment method(s) (e.g., portion size estimation, number of days and items recorded, how it was developed and administered, and how its quality was ensured). Report if and how supplement intake was assessed.
		Describe comparability of assessment methods if there is >1 group.	
			Nut-8.2. Describe and justify food-composition data used. Explain the procedure to match food composition with consumption data. Describe the use of conversion factors used, if applicable.
			Nut-8.3. Describe the nutrient requirements, recommendations, or dietary guidelines and the evaluation approach used to compare intake with the dietary reference values, if applicable.
			Nut-8.4. When using nutritional biomarkers, additionally use the STROBE-ME. Report the type of biomarkers used and their usefulness as dietary exposure markers.
			Nut-8.5. Describe the assessment of nondietary data (e.g., nutritional status and influencing factors) and timing of the assessment of these variables in relation to dietary assessment.
			Nut-8.6. Report on the validity of the dietary or nutritional assessment methods and any internal or external validation used in the study, if applicable.
Bias	9	Describe any efforts to address potential sources of bias.	Nut-9. Report how bias in dietary or nutritional assessment was addressed (e.g., misreporting, changes in habits as a result of being measured, or data imputation from other sources).
Study size	10	Explain how the study size was arrived at.	—
Quantitative variables	11	Explain how quantitative variables were handled in the analyses. If applicable, describe which groupings were chosen, and why.	Nut-11. Explain categorization of dietary/nutritional data (e.g., use of N-tiles and handling of nonconsumers) and the choice of reference category, if applicable.
Statistical methods	12	(a) Describe all statistical methods, including those used to control for confounding. (b) Describe any methods used to examine subgroups and interactions. (c) Explain how missing data were addressed. (d) Cohort study: if applicable, explain how loss to follow-up was addressed. Case-control study: if applicable, explain how matching of cases and controls was addressed. Cross-sectional study: if applicable, describe analytical methods taking account of sampling strategy. (e) Describe any sensitivity analyses.	Nut-12.1. Describe any statistical method used to combine dietary or nutritional data, if applicable.
			Nut-12.2. Describe and justify the method for energy adjustments, intake modeling, and use of weighting factors, if applicable.
			Nut-12.3. Report any adjustments for measurement error (i.e., from a validity or calibration study).
Results			
Participants	13	(a) Report the numbers of individuals at each stage of the study (e.g., numbers potentially eligible, examined for eligibility, confirmed eligible, included in the study, completing follow-up, and analyzed). (b) Give reasons for nonparticipation at each stage. (c) Consider use of a flow diagram.	Nut-13. Report the number of individuals excluded based on missing, incomplete, or implausible dietary/nutritional data.
Descriptive data	14	(a) Give characteristics of study participants (e.g., demographic, clinical, social) and information on exposures and potential confounders. (b) Indicate the number of participants with missing data for each variable of interest. (c) Cohort study: Summarize follow-up time (e.g., average and total amount).	Nut-14. Give the distribution of participant characteristics across the exposure variables if applicable. Specify if the food consumption of the total population or consumers only were used to obtain results.
Outcome data	15	Cohort study: Report numbers of outcome events or summary measures over time. Case-control study: Report numbers in each exposure category, or summary measures of exposure. Cross-sectional study: Report numbers of outcome events or summary measures.	—
Main results	16	(a) Give unadjusted estimates and, if applicable, confounder-adjusted estimates and their precision (e.g., 95% CI). Make clear which confounders were adjusted for and why they were included. (b) Report category boundaries when continuous variables were categorized. (c) If relevant, consider translating estimates of relative risk into absolute risk for a meaningful time period.	Nut-16. Specify if nutrient intakes are reported with or without inclusion of dietary supplement intake, if applicable.
Other analyses	17	Report other analyses conducted (e.g., analyses of subgroups and interactions and sensitivity analyses).	Nut-17. Report any sensitivity analysis (e.g., exclusion of misreporters or outliers) and data imputation, if applicable.
Discussion			
Key results	18	Summarize key results with reference to study objectives.	—
Limitations	19	Discuss limitations of the study, taking into account sources of potential bias or imprecision. Discuss both direction and magnitude of any potential bias.	Nut-19. Describe the main limitations of the data sources and assessment methods used and implications for the interpretation of the findings.
Interpretation	20	Give a cautious overall interpretation of results considering objectives, limitations, multiplicity of analyses, results from similar studies, and other relevant evidence.	Nut-20. Report the nutritional relevance of the findings, given the complexity of diet or nutrition as an exposure.
Generalizability	21	Discuss the generalizability (external validity) of the study results.	—
Other information			
Funding	22	Give the source of funding and the role of the funders for the present study and, if applicable, for the original study on which the present article is based.	—
Ethics		—	Nut-22.1. Describe the procedure for consent and study approval from ethics committee(s).
Supplementary material		—	Nut-22.2. Provide data collection tools and data as online material or explain how they can be accessed.

1 Reproduced from references 1 and 2 with a CC-BY license. Nut, adapted recommendations for nutritional epidemiology studies; STROBE, STrengthening the Reporting of OBservational studies in Epidemiology; STROBE-ME, STROBE Extension for Molecular Epidemiology; STROBE-nut, STROBE for nutritional epidemiology studies.

The aim of the present Perspective is to further explain the rationale for and elaborate on the items of the STROBE-nut recommendations to enhance the clarity and to facilitate the understanding of the recommendations. The main target group of STROBE-nut consists of researchers working with observational studies of diet and health. The checklist can also be of use to reviewers and editors, as well as to researchers working with dietary assessment in other contexts. Information on how to design studies, select methods for dietary data collection, or how to handle and analyze dietary intake data is available in textbooks and websites developed for these purposes.

Published examples that show how to report some aspects of each item comprehensively are provided in the running text (and as **Supplemental Examples** with **Supplemental References**), but these do not necessarily imply that the cited study was well reported overall or had a higher quality than other studies. Some examples have been slightly edited to conform to current Journal style. In Text Box 1 and **Text Boxes 2****–****9**, theoretical background information is presented.

**TEXT BOX 2 DIETARY ASSESSMENT METHODS**

The most common dietary assessment methods in use today are the retrospective FFQs, 24-h recall interviews, and prospective food records, all of which rely on self-reports of dietary intake. Each dietary assessment method has its own strengths and limitations, and the suitability of the different methods depends on the purpose of the study.

Although dietary assessment methods are useful tools to assess intake, no perfect measure of diet exists. It has been shown in validation studies that used unbiased biomarkers that self-reported energy intakes are not equivalent to true intakes (14, 15). Consequently, Subar et al. (16) pointed out that energy intake estimates per se are not suited to make inferences about disease outcome. Nevertheless, dietary assessment methods have proved to be useful tools to examine associations between relative (energy-adjusted) dietary intakes and disease outcomes (16, 17). Below is a short description of each method’s main characteristics.

The FFQ is the most commonly used method in today’s large-scale epidemiologic studies, designed to provide individual information on the habitual diet and often intended to cover the past 6–12 mo. It was developed to enable the rank ordering of the participants’ dietary intakes (17) and is based on a list of specific foods together with multiple response categories on how often each food is consumed. To accurately capture a population gradient, an FFQ must include food items commonly consumed in the population and present relevant frequency options.

The frequency with which a food item is consumed is considered to be the main factor influencing the ability to rank individuals on nutrient intakes (18–20). This might be explained by a larger variation in portion sizes within-person than between-person (17) and by the participant’s ability to more accurately report habitual frequencies than habitual portion sizes (21). Questionnaires that estimate frequencies in combination with portion size assessments have, however, been shown to improve the ranking of individuals according to intakes of energy and nutrients compared with those with no portion size estimation (22). Estimates of portion sizes can be based on questions in the questionnaire, by predetermined standard portions, or by a combination of these alternatives.

The number of included food items will affect the ability to capture the habitual diet. Longer FFQs (i.e., with a large number of food items) tend to produce better ranking of “usual” intakes of energy and several nutrients (23, 24). Longer food lists, however, have a tendency to give higher, potentially exaggerated, estimates of absolute intakes (25, 26). Therefore, FFQ-derived estimates of dietary intakes need to be examined in relative terms (i.e., energy-adjusted; see Text Box 4). Research has shown that when principles of cognitive psychology are followed in method development, long FFQs may be easier to complete and will potentially provide more accurate dietary estimates (21, 27, 28). However, short FFQs or so-called screeners may successfully rank individuals on specific foods or particular nutrients found in certain foods (29).

The retrospective 24-h recall and the prospective food records provide detailed dietary reports of the current diet at the individual level. In the 24-h recall method, the participant is interviewed about the consumption the previous day and the food record method that is used to record intake in a diary at the time of consumption. The 24-h recall is affected by the ability to recall what was eaten yesterday, whereas the food record itself may affect the intake during the registration.

Single recalls or records say very little about the individuals’ habitual diet, but they provide good estimates of the mean intakes of groups (30). To enable the rank ordering of individuals and to obtain an approximation of the usual diet, repeated recalls or records (from the same individual) are required (31), although the number of days needed differ by nutrients and population groups (i.e., depending on the intraindividual variation in intake). Repeated food records or 24-h recalls are the preferred methods to describe the intake distribution in a population and the proportion of the population at risk of inadequate intakes, or below or above recommended intakes. A combination of repeated 24-h recall and FFQ data may provide data superior to the use of either method alone (14, 32), especially for foods that are not regularly consumed. Such an approach would resemble the dietary history methodology, which has the aim of assessing the usual or habitual intakes in individuals.

The dietary history method was described already in 1947 by Burke (33). It consists of a meal-pattern interview, accompanied by a food list with questions on usual frequencies and portion sizes of foods and 3 self-administrated food records. The information obtained with the food records and the food list serve as cross-checks to clarify the information obtained in the meal pattern interview. The method, which today exists in many varieties, has the potential of providing very detailed information but is time-consuming and expensive. Over the years, several adaptations and modifications have been undertaken, but the interview-administered dietary history is generally not suited for large-scale studies. One advantage of the methodology is the combination of different types of dietary data (i.e., both the habitual and actual or current diet).

Dietary exposure assessment is an active field of research in which new or improved dietary assessment methods (34, 35) and ways to combine dietary data (36–38), adjust for measurement error (39), and aggregate intake data through statistical intake modeling (40–43) are being developed almost continuously. In addition, newly emerging information and communication technology used for dietary assessments have been characterized (44). These methodologies need to be validated and clearly reported to enable reproduction and adaptation in other settings. In addition, regardless of which dietary assessment method is being used, any assumptions, limitations, or statistical modeling that may introduce systematic or random errors should be documented and reported.

Definitions and terminology to describe traditional methods used to assess dietary, food, and nutritional intake have been provided previously (17, 28, 45). For researchers seeking information on the best approach to dietary assessment, there are currently a number of Internet sites available that provide useful resources describing different relevant methods [e.g., the United Kingdom Medical Research Council’s toolkit for diet and physical activity measurements (46) and the Dietary Assessment Primer of the National Cancer Institute, NIH, United States (47)] as well as websites providing access to specific tools [see (48)].

**TEXT BOX 3 MISREPORTING**

Misreporting of dietary intake is a major challenge when examining the association between dietary factors and health. Underreporting of energy intake, a more extensive problem than overreporting, tends to be related to personal characteristics such as overweight, obesity and weight consciousness, sex, age, socioeconomic factors, psychological traits, and psychosocial and behavioral factors (51–56). Reported low energy intakes in overweight and obese individuals might also be a consequence of dieting during dietary assessment (57–59).

Some studies suggest that energy underreporting may be selective by affecting fat and sugar intakes to a greater degree (52, 60). Even when energy intake estimates agree with energy expenditure, differential under- or overreporting of specific foods may still introduce bias in the interpretation of dietary intakes, and potentially influence both macro- and micronutrients (52).

Reports of habitual energy intake can be evaluated by assessing if such values are able to cover the physiologic energy requirements of the subjects in a study (61, 62) (see Text Box 8). This kind of evaluation can be carried out by using the complete population or by using appropriate subgroups (e.g., men and women). It requires estimation of a value for the physical activity level (PAL), appropriate for the activity level and lifestyle of the population under evaluation. The WHO has provided PAL values for different categories of physical activity (63). Black (61, 64) recommended that subjects in epidemiologic studies be classified into low, medium, and high PAL values as a possibility to improve the identification of gross bias due to underreporting across the full range of energy requirements. The evaluation also requires an estimate of basal metabolic rate (BMR) appropriate for the population. Equations for predicting BMR on the basis of sex, age, weight, and height are available (63, 65), making estimates possible without a measuring procedure. The food intake level (FIL) is then calculated as energy intake divided by BMR (62). For subjects in energy balance, FIL should equal PAL.

A comparison of FIL and PAL values can serve as a useful screening procedure to evaluate if the reported energy intakes are reasonable. The comparison is applicable for the majority of healthy individuals, including children >2 y of age (i.e., special considerations are needed for pregnant and lactating women) (66). However, as described above, this evaluation procedure requires several assumptions, which may limit its accuracy. Populations with a very high prevalence of overweight and obesity may represent a concern, because equations to predict BMR on the basis of body weight tend to be inaccurate for subjects having a large proportion of adipose tissue, which has a lower metabolic rate than lean tissue (67). In such populations, it may be appropriate to use measured rather than predicted BMR.

**Missing consumption frequencies in FFQs.** Studies that examined the nature of unanswered items in FFQs have shown that the response category is more likely to be left blank for foods eaten never or seldom (68–70), and the proportion of “true” nonconsumption is higher for these foods and lower for more widely consumed food items (69, 71). In studies in which missing values were imputed with a null value, the estimated mean intake of energy and nutrients was observed to decrease by the number of missing values (68, 72), indicating that missing values may both be systematic (i.e., nonconsumption) and random. Therefore, any method to replace them is problematic (e.g., by imputing with a null value or with the median or mean values from other participants, or by using multiple imputations), but currently no other alternative is available.

Overall, misreporting in dietary assessment is a complex issue to handle. Its significance is best examined in sensitivity analyses, examining subgroups with implausible data separately (see Text Box 8). However, individuals with extreme values, which results in intake data that are not compatible with biological function (17), are another matter. In large epidemiologic studies this usually affects less than a few percent, and it is advised to exclude these individuals from analyses.

**TEXT BOX 4 ENERGY ADJUSTMENTS**

Energy intakes (i.e., absolute intakes) based on self-report methods are often poorly measured, although the degree of misreporting varies between different dietary assessment methods and subjects, and self-reported energy intakes should therefore not be used as exposure variables (16). There are 2 main reasons for energy adjustment of food and nutrient intakes. First, the amount of food needed differs depending on body size, physiologic status, PAL, and metabolic efficiency (17, 80) [see also the Dietary Assessment Primer (47)]. By using energy adjustment, intake data are evaluated at an isocaloric level in line with the concept that the composition of the diet, independently of total energy intake, is of primary interest in relation to disease risk. Individuals with high energy intakes tend to have higher consumption of most nutrients, and failure to adjust nutrient intakes for energy intake can lead to misleading conclusions. Second, because the errors in reported intakes of energy and other food components are correlated with each other, it is recommended to use self-reported energy intakes to adjust other self-reported dietary components for measurement error (81). That is, energy adjustment will reduce the artificial interindividual variation introduced by under- and overreporting of food intake, and some of the negative influence of dietary measurement error will be removed. It is generally accepted that energy adjustment is advantageous in analyses of diet-disease associations and therefore nearly always used in nutritional epidemiology (81). Validation studies have also repeatedly shown that FFQs provide more reliable information on nutrient intake when examined in relative terms as compared with the absolute intakes (14, 15).

The most common methods to adjust nutrient or food intakes are the residual method and the nutrient density method. In the residual method, energy-adjusted intakes are the residuals from a regression model with total energy intake as the independent variable and nutrient intake as the dependent variable (80). With the nutrient density method, macronutrients (protein, carbohydrate, fat, and alcohol) are expressed as proportion of energy (percentage of energy), whereas micronutrients or food groups often are expressed as intake per 1000 kcal or intake per mega-Joule.

When total energy intake is believed to be an important predictor of disease, the model estimating disease risk should include both the energy-adjusted nutrient variable (i.e., the residuals) and the total energy intake. In populations with a large variation in body weight and lean body mass, as well as in comparisons between sexes, nutrient densities are especially useful. However, the nutrient density method may introduce a spurious inverse relation between nutrient and energy intakes. Therefore, it is recommended to also include total energy intake in the multivariate nutrient density models of disease risk, because this will examine the nutrient composition (i.e., nutrient density) of diet and also control for the confounding by energy intake (17). This adjustment makes the nutrient density and residual methods comparable when assessing associations between food intake and disease.

**TEXT BOX 5 RANDOM AND SYSTEMATIC ERRORS IN DIETARY ASSESSMENT**

Measurement error in dietary assessment may have many origins, be present in various degrees, and may either be random or systematic. A number of Internet sites provide resources describing this (46, 47), as well as websites providing access to specific tools (48).

Nutritional epidemiology studies often aim to provide an accurate estimate of the “usual” habitual diet. This is a challenge because human diets are prone to large day-to-day variations, resulting in random errors in the dietary assessments. Random errors may also be associated with the specific dietary assessment tool, its administration, or inconsistencies within the individual. These problems may partly be overcome by selecting an appropriate methodology, a carefully designed tool, and by using standardized instructions and procedures.

A larger number of days (i.e., 24-h recalls or food records) per individual will reduce the variation within individuals. Repeated administrations of an FFQ may improve estimates by capturing changes in dietary habits over time in a cohort study. However, errors associated with the FFQ format or inconsistencies in individuals are difficult to specify and estimate and cannot be rectified by simply increasing the number of administrations per individual (84). Random errors in dietary assessments may result in attenuation of diet-disease associations, which needs to be considered in the interpretation of null associations (85). The precision (i.e., the relative absence of random errors in the measurements) can generally be improved by increasing the sample size of the study, irrespective of the dietary assessment method used.

Nonrandom, systematic error (i.e., bias) is a condition that causes the measurement to depart from the true value in a consistent direction (4, 86). Systematic errors are problematic, because such errors could cause erroneous conclusions about the distribution of dietary intakes or the associations between nutritional exposures and health outcomes (87). Two main types of systematic errors are information bias and selection bias, where the latter refers to the systematic error that derives from the sampling procedure or self-selection due to nonresponse or systematic drop-out and may occur in non–population-based case-control studies or in cohort studies with incomplete follow-up.

Information biases of specific relevance in nutritional epidemiology are systematic errors during data collection (measurements of diet and covariates) that lead to wrong conclusions about dietary intakes or diet-outcome associations. For discrete variables, such measurement error is often referred to as misclassification. Differential misclassification is serious when classification differs according to outcome status. Nondifferential misclassification may lead to attenuated associations (i.e., bias toward the null) if the exposure is on a dichotomous scale, such as when exposed individuals are compared with unexposed. In contrast, with polychotomous categorization (e.g., quintiles), which is common for dietary exposures, there is a danger that bias away from the null will appear (i.e., nonexistent associations are created) (88). Similarly, dietary data analysis that uses energy-adjustment models, in which correlated errors in the dietary variables may be present, could result in biased exposure effects of arbitrary size and direction (87).

Erroneous or distorted reports of dietary habits can be linked to the format of the dietary assessment tool, the underlying database, to the study participant’s interaction with the assessment method, or to the interaction between the interviewer and interviewee. One example is that the study participants report intakes believed to be socially acceptable or in line with the prevailing recommendations (i.e., social desirability bias). Another example is recall bias (i.e., when the reported diet is influenced by the participant’s knowledge of the diagnosis), such as if cases remember and recall their previous exposure in another way than controls. If all participants are free of disease at baseline (i.e., cohort studies), the misclassification of exposure is most likely nondifferential in relation to the disease, but could still depend on other factors present at baseline (see Text Box 3).

Subgroups with certain diet-related diseases or those with potentially under- or overreported energy intakes may be considered for exclusion. An alternative approach is to examine the robustness of study findings separately for subgroups with potentially dubious dietary reports.

Although a representative study sample is considered a requirement to extrapolate study conclusions about dietary exposure to the general population, the absence of statistical representativeness, based on sampling from a source population, does not prohibit researchers from drawing conclusions about diet-disease associations. Instead, internal validity with a low degree of systematic error is of crucial importance in etiologic epidemiology. The restriction of participants may be a way to prevent confounding (89). Furthermore, the estimates of associations might be unbiased, even if the prevalence estimates of dietary exposure are biased due to (self-)selection of participants (90).

**TEXT BOX 6 VALIDITY AND REPRODUCIBILITY IN NUTRITIONAL EPIDEMIOLOGY**

Because measurement errors arising from the assessment of dietary intakes may have a crucial impact on study results and conclusions, it is of fundamental importance to evaluate the validity of the assessment method (4). The validity is best assessed by using >1 approach (17, 39, 91). Because the validity of an instrument may differ between populations, internal validation (i.e., performed within the population studied) is the standard approach. In addition, measurement errors may differ between different dietary variables (e.g., energy, foods, and nutrients) within a study (14, 60). It is therefore important to evaluate the validity of several aspects of the diet. The concept of energy adjustment (see Text Box 4) also applies to validation studies.

Biomarkers have the advantage of providing an objective assessment of an instrument’s ability to assess the “true” habitual intake. Three types of validation biomarkers with different uses are available: recovery, predictive, and concentration biomarkers (see Text Box 7). At present, only a limited number of biomarkers are available, which compromises the possibility to evaluate all aspects of a dietary assessment tool, but the knowledge about biomarkers and their use is rapidly increasing (92–95).

As a complement to biomarkers, or when biomarkers are not available or feasible, the relative validity of one dietary assessment method can be evaluated by comparing the results with those obtained by means of another (i.e., a reference method). In studies that evaluate FFQs and dietary history methodologies, repeated food records or 24-h recalls are common reference methods.

A larger number of records or recalls (i.e., covering daily and seasonal variability in dietary intake) give a higher precision of the reference method. The relative evaluation of 2 methods with the same measurement error may, however, give a false impression of acceptable coherence and validity. As a rule of thumb, for a relative validation of an FFQ, weighed, repeated food records are preferred over estimated repeated records or 24-h recalls. This is because the portion sizes are weighed and not estimated, and because the prospective reporting of food consumption is less dependent on memory than the retrospective reporting in an FFQ.

The overall bias of a method (i.e., under- or overestimation of dietary intake) can be shown by group mean differences, by the outcome from a Bland-Altman plot (96), or (for energy) by comparing energy intakes with total energy expenditure (Text Box 8). The Bland-Altman method (96) estimates the agreement between 2 methods and indicates whether the results differ depending on the size of the values. In the context of nutritional epidemiology, the Bland-Altman method assumes that estimates reflect absolute dietary intakes and is therefore more suitable for examining the validity of repeated records or recalls. FFQs are primarily designed to rank-order dietary intakes and are therefore less accurate in estimating absolute intakes (14, 15). Correlation, regression, and Bland-Altman plots cover different aspects of validity and can be used as appropriate measures, reported together (39). When data are categorical or simply yes or no, other methods are used (e.g., κ, sensitivity and specificity).

The partial correlation analysis allows adjustment for major confounding factors in a validation study (97). Because the analysis describes a dose response, it could be interpreted as a measure of attenuation (i.e., provide some indication on whether the estimated relative risk of disease is likely to be attenuated by using the tool), which is helpful for researchers when interpreting and discussing observed associations. In addition, information about the degree of attenuation will help researchers when preparing for future studies to estimate the potential loss of power and the necessary sample size.

Reproducibility (or reliability) refers to the consistency of a measure, such as when a questionnaire is administered repeatedly to the same persons at different time points or when the agreement between assessors is evaluated (e.g., through a comparison of 2 observers’ estimations of portion sizes). The strength of an agreement can be expressed through the intraclass correlation coefficient, as the proportion of the between-person variance to the total variance (i.e., the sum of both within- and between-variation) (98) (see also Text Box 7).

The presence of exposure measurement error and misclassification in nutritional epidemiology has led researchers to investigate how to use data from validation studies to try to correct for biases when examining associations between dietary exposures and disease risk in large-scale epidemiologic studies (81, 99–101). Statistical methods have been developed and the statistical field has grown (102). The fully multivariate regression calibration method takes measurement error into account, when the validation study previously has evaluated the dietary assessment method against a valid standard method (97, 99–102). Including a range of potential confounders enables the estimation of both attenuation and contamination factors. Because these statistical methods are all based on specific assumptions, the reports clearly need to be comprehensive to ensure a balanced interpretation (see Nut-12.3). However, this approach will not compensate for weak instruments or an overall poor validity (see Nut-19).

**TEXT BOX 7 NUTRITIONAL BIOMARKERS**

Nutritional biomarkers are used as objective markers of dietary exposures. They are unbiased by self-reporting aspects of intake assessment methods and are often measured in blood or urine. Nutritional biomarkers can be used in studies examining the validity of dietary assessment methods, as measures of compliance in intervention studies, and to examine associations with disease outcomes [see also STROBE-ME (107)]. They are also useful when the information in food-composition databases is missing or unsatisfactory with regard to certain compounds (e.g., *trans*-FAs, phytoestrogens, and acrylamide). In addition, to combine appropriate biomarkers with dietary intake data in a cohort study may strengthen the statistical power to detect diet-disease associations (106).

There are 3 main types of nutritional biomarkers; recovery, predictive, and concentration (93, 108). Recovery biomarkers give estimates of absolute intakes within a specific time period and can assess the degree of misreporting in dietary data assessed in parallel. Because they often are less suitable for use in large populations, they are mainly applied in validation studies. The doubly labeled water (DLW) method estimates total energy expenditure and is applicable in a wide range of human subjects, including premature infants, pregnant and lactating women, and elderly people. In the DLW method, subjects consume water labeled with the stable isotopes deuterium and oxygen-18 and are required to provide samples of body fluid (most commonly urine) before and after drinking the dose. Samples are generally collected during 1–2 wk. Useful guidelines with regard to appropriate procedures, quality control, and recommendations for presentation of results are available (109).

The 24-h urinary excretion of nitrogen is used to assess protein intake (4). One 24-h urine excretion/subject is sufficient for the calculation of protein intake of groups, but ≥8 consecutive 24-h collections are needed to assess the protein intake of an individual (110). Para-amino-benzoic acid (i.e., 4-aminobenzoic acid) is a reliable marker that can be used to estimate the completeness of urine collections (108).

The 24-h urinary excretion of sodium and potassium is also useful and represents an inexpensive recovery biomarker for sodium and potassium intake, respectively. Especially when assessing the intake of table salt, biomarkers are valuable; the amount consumed varies greatly, because salt can be added to food products during the manufacturing process, during food preparation, and by the individual during meals.

Predictive biomarkers show high dose-dependent correlations with true intakes, but the recovery is incomplete. Once the difference has been estimated (through feeding studies) and the biomarker is calibrated, it can be used as a reference instrument (111, 112). An example is combining 24-h urinary sucrose and fructose, which is closely related to the intake of total sugars, whereas only a very small fraction of the sugars ingested is present in urine. Predictive biomarkers provide information that ranks between recovery and concentration biomarkers with respect to the extent they can objectively indicate dietary exposures.

Concentration biomarkers are correlated with dietary intakes but are unable to quantify the absolute intakes. However, because they are correlated with dietary intakes, they are suitable for ranking individuals on dietary exposures (113). Some examples of concentration biomarkers measured in various tissues are carotenoids, polyphenols, and vitamin C as markers for fruit and vegetable intakes; isoflavonoids for soy intake; alkylresorcinols for intakes of whole grain of wheat or rye; and various FAs for the consumption of dairy products and fish.

The ideal nutritional biomarkers should, in addition to accurately reflecting dietary intakes, be specific for the particular food or nutrient, sensitive (i.e., showing a dose-response relation), stable, not too expensive, easy to measure, and also identified as either reflecting long- or short-term intake. Epidemiologic studies are usually interested in assessing long-term intakes. Samples from hair and nails reflect a time period of months or years, whereas urine, feces, and plasma samples may reflect a shorter period. It has been shown that three 24-h urinary samples are sufficient to measure long-term exposure for a range of biomarkers (114). The ability to reflect the dose-response association in a population, and the reliability of a concentration biomarker, is identified by the intraclass correlation coefficient (17, 115) (see Text Box 6).

Although biomarkers are unaffected by self-reporting, there are physiologic factors that contribute to between-person variability in concentration biomarkers influencing the correlations between biomarkers and dietary exposures. These physiologic factors include variations in absorption, metabolism, and excretion as well as the influences of lifestyle factors and microbiota (113). In addition, the type of biological material, the sampling method, and the choice of analytic method influence the measured concentrations. The understanding of how metabolism and disease are affected by diet, nutrients, and genetics is an emerging field (95).

Improved instruments and analytical strategies in metabolomics have made it possible to measure many compounds simultaneously in a sample (92). Metabolomics has been shown to be a promising tool for the discovery of novel nutritional biomarkers in the human metabolome. For example, novel biomarkers can be found in the “food metabolome,” the part of the metabolome directly derived from the digestion and biotransformation of foods and their constituents (116).

**TEXT BOX 8 ENERGY BALANCE AND PHYSICAL ACTIVITY**

The concept of energy balance is based on the first law of thermodynamics, which states that energy can be transformed from one form to another but cannot be created or destroyed. This is useful when evaluating energy intake data because any change in body energy content over a specific time period is equal to energy intake minus energy expenditure during the same period of time: the so-called energy balance equation (118). For groups of people, it is valid to assume that if the average body weight of the group is constant over time, this group is in energy balance.

The energy metabolism of human subjects varies within a 24-h period, and across the life span, reflecting fuels metabolized for maintenance, tissue synthesis, physical activity, lactation, etc. The main components of total energy expenditure (TEE) in human subjects are as follows: the resting energy expenditure or the BMR, the thermic effect of food, and the physical activity energy expenditure (118). BMR represents the energy expenditure at rest under controlled conditions and is generally measured by using indirect calorimetry.

The energy content of foods in the diet is not completely available to the body because some energy is lost in feces and urine. The metabolizable energy of foods, as given in food-composition tables, represents the energy content (i.e., from macronutrients and dietary fiber) available to cells for conducting biological processes.

The DLW method can be used to evaluate the energy intake of human subjects in energy balance. It can be used to measure TEE and has the advantage that it estimates energy expenditure during free-living conditions in subjects without causing any important changes in their behavior (109) (see also Text Box 7). It is more complicated when energy balance cannot be assumed—for example, during growth, weight gain, or lactation. However, the deviation from energy balance is often small and unimportant; and on the basis of knowledge of human physiology, it is often possible to correct for such deviations.

Physical activity is a variable of interest in many epidemiologic studies because it is associated with physical fitness and health, and because it is a component of TEE (119). Although BMR often constitutes the main part of TEE, energy expended in response to physical activity is the most variable part. Physical activity is often expressed as PAL calculated as TEE divided by BMR (see also Text Box 3). Thus, physically active subjects will have a high PAL value, whereas a low PAL indicates inactivity. An advantage of the PAL concept is that it provides an estimate of physical activity, which is independent of body weight. It is also possible to assess the ratio between the energy expended when performing a specific activity and an estimate of the resting energy metabolism, so-called metabolic equivalent of task (MET) values or physical activity ratio values. Compilations of MET values for a large number of activities are available (120, 121). Such values are sometimes used to describe the pattern of physical activity (see Nut-8.5). However, published MET values may introduce bias if used to calculate PAL or TEE, because they assume a value for resting energy metabolism that is too high for many individuals (122).

As a confounding factor, physical activity is often of interest in studies of diet and health. It may also be associated with disease risk in itself. Physical activity is a multidimensional behavior with many characteristics, such as type of physical activity, frequency, duration, and intensity. It can be studied by using a variety of methods: for example, diaries, questionnaires, pedometers, heart rate monitoring, or accelerometry (119). The choice of method or methods is dependent on the aspect of physical activity in focus. Ainsworth et al. (120, 121) provided recommendations on how to assess physical activity by means of self-report. Currently, there is no consensus with regard to the best way to evaluate methods intended to assess physical activity, and consequently all estimates of this variable are associated with some degree of uncertainty.

**TEXT BOX 9 FOOD DATABASES AND CALCULATION OF DIETARY DATA**

Studies aiming to examine the intakes of energy, nutrients, and other food components require reliable data on food composition to convert intakes of food into intakes of nutrients and other food components. The quality of the food-composition data may differ, and because of differences in food use across countries (e.g., variety, soil, processing, and fortification), the nutrient values reported by most national and regional databases are not readily comparable at an international level. A number of other artificial differences may also occur as a result of component identification, food description and nomenclature, analytical methods, mode of expression, and units used (126).

The objective of the dietary exposure assessment should be carefully considered before making any decisions with regard to food-composition data. This is particularly important in multicenter studies that involve more than one country, or when the objective is to examine nontraditional exposures (e.g., food additives or pesticide residues). In longitudinal studies, care should be taken with regard to changes in food composition per se, as well as changes in the chemical analyses of food composition. Any changes require careful thought before the decision on which specific food database or food-composition data set to select. The geographical area and eating culture, and an assurance that foods typical of the region are included, are important criteria when selecting a food-composition table. However, if local data are too limited with regard to food items or food components, or if the quality of the data is uncertain or insufficient, then the food-composition data from neighboring countries may be more appropriate.

Although no formal quality system is in place for food data compilation, the overall aim of the International Network of Food Data Systems (127) is to stimulate and coordinate efforts to improve the quality and availability of food-composition data worldwide. On a European level, The European Union’s FP6 and FP7 European Food Information Resource (EuroFIR) (128) Network of Excellence (2005–2010) and EuroFIR NEXUS (2011–2013) projects aimed to standardize and harmonize food-composition data in Europe through improved data quality, database searchability, and standards. Recommendations for food description, component identification, value documentation, recipe calculation, quality evaluation of values, guidelines to assess analytical methods, document and data repositories, and training opportunities were harmonized as elements of this EuroFIR quality framework.

Another important, often challenging, step when assessing intakes of food components is the matching of the consumed food with a food item in the database. The matching procedure is critical for obtaining high-quality estimates of dietary exposures. Enhancing databases with brand-level information could help avoid this limitation (35). Matching errors in food-composition data may occur when one unit is converted into another (e.g., converting servings or ounces to grams) or when the conversion uses a specific denominator or expression. The handling of missing data and values is another challenging step in the matching procedure. All food items reported in the dietary assessment may not be available in a food-composition table. Even if the particular food is available, certain nutrients may not be well covered by the food database. The need for, and availability of, conversion factors to be applied to the consumed food amounts (e.g., raw-to-cooked conversion) and the appropriate concentration of food components (e.g., nutrient retention, yield, or bioactivity) are other challenges. The FAO International Network of Food Data Systems (INFOODS) has developed guidelines to assist researchers wishing to match food consumption data with the most appropriate items in food-composition databases (129).

When the aim is solely to assess the exposure distributions of environmental hazards (e.g., pesticide residues in foods or food contaminants), “deterministic” or “probabilistic” approaches can be used. The deterministic approach, which calculates exposure as the product of a point estimate of the component in the food item (e.g., the average, mean, or maximum concentration) and the amount of the food item consumed during a specified time period, is the most common. Because this approach assumes that the exposure is fixed and precisely known, which, in reality, rarely is the case (130), the result may be a “piling up” of worst-case assumptions with a very low probability of occurring. The probabilistic approach, in contrast, uses distributions instead of fixed values. This makes it possible to include variation and uncertainty in the calculations, and therefore to show a range of possible results and the probability for each of the results.

Because foods are consumed together, and nutrients and other food components are concentrated in certain foods, the study design, statistical methods, and interpretation should consider these dependencies. In addition, the limitations in consumption and concentration data need attention. These include uneven coverage of data, large proportions of censored data, and uncertainties about the shape of the distribution.

## The STROBE-nut Checklist Items

The STROBE-nut includes checklist items (presented as Nut) organized according to the different sections usually included in scientific articles: title, abstract, methods, results, discussion, and complementary materials. All areas should be addressed in an article, but the location and order may vary according to the specific journal guidelines. Some of the original STROBE items (6) were considered sufficient also for nutritional epidemiology articles, and explanations and elaborations of these items can be found in the article by Vandenbroucke et al. (3). This means that some of the STROBE-nut checklist numbers appear to be missing; for instance, there are no items Nut-2, -3, or -4. Further explanations for all specific items listed in the STROBE-nut checklist are shown below.

### Title and abstract

#### Nut-1. State the dietary and nutritional assessment method(s) used in the title, abstract, or keywords.

##### Example 1.

“The consumption of sugar-sweetened beverages was derived from 7 repeated FFQs administered between 1980 and 2002” (9).

##### Explanation.

Reporting the dietary and nutritional assessment method or methods in the title, abstract, or keywords with accurate terminology contributes to the completeness of the manuscript (10). This may be particularly relevant for methodologic research articles, which are used as reference articles in association studies. In addition, it will facilitate the accuracy of indexing in electronic databases as well as ease literature searches, through the use of keywords (11, 12).

Due to the growing number of scientific journals, indexing of articles increasingly applies both automated summaries and manual approaches (11). If reports from dietary or nutritional research use standard terminology or approved Medical Subject Headings (MeSH) (12), a step is taken toward reducing the number of incomplete or unusable research reports (13). Readability should be ensured at all times, and journal specifications with regard to style and word count apply. Guides to appropriate terminology can be found online (see Text Box 2).

### Methods

#### Nut-5. Settings: describe any characteristics of study settings that might affect the dietary intake or nutritional status of the participants, if applicable.

##### Example 1.

“In a Matlab area, an embankment was constructed between 1982 and 1989 on the banks of the rivers Meghna and Dhonagoda to protect the area from seasonal floods. The study villages are therefore also categorized in relation to whether they are situated inside or outside the embankment. This embankment has a great impact on the pattern and production of major crops and fish on both sides and is believed to have an effect on food availability and consumption, which, in turn, could lead to effects on nutritional status” (49).

##### Explanation.

Clear information about the study setting is needed to facilitate the interpretation and generalization of the findings (see Text Box 2). This includes external conditions that may affect dietary intake or nutritional status of the population, as well as the reporting of these. The time frame for the dietary assessment is also an important factor. Etiological studies mostly focus on dietary intakes over longer time periods, rather than intake during a certain day or week. Because the day-to-day variation as well as the seasonal variation, including holiday periods and special events, may influence observed estimates of habitual intake, the time period covered should be outlined. When using short-term dietary assessment methods, information is required with regard to the time period between examined days, and how weekdays and weekends are covered.

#### Nut-6. Participants: report particular dietary, physiologic, or nutritional characteristics that were considered when selecting the target population.

##### Example.

“Nonsmoking women, 20–50 y of age, not occupationally exposed to cadmium, were recruited. Women were chosen as subjects because they have higher cadmium concentrations in blood and higher body burdens of cadmium than men. Furthermore, low iron stores, which have been associated with increased gastrointestinal absorption of cadmium, are more common among premenopausal women. Because cigarette smoking may significantly increase body burden (kidney concentration) and blood cadmium concentration as much as 5 times, only women who had been nonsmokers for ≥5 y were eligible for the study. None of the women were pregnant or lactating at the time of the study” (50).

##### Explanation.

Because of the potential influence on study results and generalizability, eligibility and exclusion criteria related to dietary intake or nutritional status are especially important to report in nutritional epidemiologic studies. Such characteristics include age, sex, smoking, BMI, and physiologic status (e.g., pregnancy). Other factors (e.g., physical activity) or conditions (e.g., disease diagnoses or obesity) that may result in dietary changes or potential misreporting of energy intake also require clear descriptions (see Text Box 3).

#### Nut-7.1. Variables: clearly define foods, food groups, nutrients, or other food components.

##### Example 1.

“The definition of whole grains applied in the current study was in accordance with that of the American Association of Cereal Chemists and is as follows: “Whole grains shall consist of the intact, ground, cracked or flaked caryopsis, whose principal anatomical components—the starchy endosperm, germ, and bran—are current in the same relative proportions as they exist in the intact caryopsis.” Cereal species investigated in the current study were rye, wheat, oats, barley, rice, millet, corn, and maize (dried); triticale; and sorghum and durra. Whole-grain intake was expressed by the following 2 different methods to calculate intake: *1*) intake of whole-grain products (grams of product per day) was calculated and consisted of 4 product categories that contained either solely whole-grain products (rye bread, whole-grain bread, or oat meal) or were dominated by whole-grain products (>75%; crispbread); *2*) to quantify the absolute amount of whole grain consumed, total whole-grain (grams of whole grain per day) intake was calculated” (73).

##### Explanation.

To assess the health benefits of a specific dietary exposure, and to compare findings across studies, it is essential that the examined dietary exposures are clearly defined. Food security indicators or measures should be clearly described when used as proxy for or an indicator of dietary intake. When the exposure variables are food groups, the components of each aggregated food group should be clearly described. When assessing the health properties of specific food items, it is helpful to specify the scientific or taxonomical names of foods, because the nutritional composition of food is strongly related to species, cultivar, and variety (74). The units used should be clearly presented (e.g., servings per day, grams per day, and liters per week). In reports of complex dietary exposures, it is helpful to use standardized approaches (if available) that uniformly describe, classify, and quantify exposures. For example, recommendations for reporting whole-grain intake in observational and intervention studies have been published (75).

In some circumstances, a high level of detail may be justified. Thus, it may be helpful to indicate recipes and report whether food intake was based on raw or cooked foods (i.e., food preparation method). In addition, the report should include how food intakes were converted into nutrients or food components by specifying the units, method of calculating intakes, and the food-composition database (see also Nut-8.2). When relevant, the full definition of non-nutrient food components (e.g., chemical form of the compounds), and the units, should be provided. Similarly, information on the method of the biochemical analysis and relevant documentation is helpful.

#### Nut-7.2. Variables: when using dietary patterns or indexes, describe the methods to obtain them and their nutritional properties.

##### Example 1.

“We performed exploratory factor analysis to extract patterns that we then confirmed by using confirmatory factor analysis. To avert subjective influences in food grouping, we included all individual food items in the exploratory factor analysis. We considered eigenvalues >1.0, interpretability of factors, and number of items and their frequency to decide how many factors to extract from the data and confirm. We included items with factor loadings of ≥0.20 from exploratory analysis to test specific factor structures by using confirmatory factor analysis; the goodness-of-fit index was high (0.93 for the model including all patterns). Factor scores were calculated for each individual for each pattern by weighting the standardized intakes of the food items by their factor loadings and summing for all items. The scores of each dietary pattern were categorized into quintiles. We derived 4 major dietary patterns: “healthy” (vegetables, fruit, and legumes), “Western/Swedish” (red meat, processed meat, poultry, rice, pasta, eggs, fried potatoes, and fish), “alcohol” (wine, liquor, beer, and some snacks), and “sweets” (sweet baked goods, candy, chocolate, jam, and ice cream)” (76).

##### Explanation.

Dietary pattern analysis allows researchers to examine total diet, or combinations of many food components, rather than single nutrients or foods. Dietary patterns can be estimated by statistical data-driven techniques (a posteriori) (77) or by dietary indexes or scores that are hypothesis based (a priori) (78). Data handling and analysis involve many steps that need to be described clearly in order for others to fully understand the procedure and to interpret findings (see also Nut-12.1).

The dietary patterns identified from the data-driven techniques are meant to reflect the dietary habits in the population independent of any previous knowledge about dietary influences on health. The most widely used data-driven approaches are cluster, principal components, and factor analysis. Reduced rank regression is another approach that uses both dietary data and a set of response variables (e.g., plasma concentrations of disease markers) to identify patterns (79).

Each of these methods has its specific procedures, and researchers are required to make several informed decisions during data handling and analysis. In order for other researchers to fully understand the procedure and to interpret findings, the report should include information on the following: *1*) the selection and aggregation of dietary variables, *2*) any standardization used, and *3*) any approach of energy-adjustment (see Text Box 4). The basis to determine the number of patterns (e.g., correlation or covariance matrices and factor loadings) and the selection criteria should also be presented. A description of the rationale for labeling the dietary pattern, as well as the nutritional properties of the emerging patterns, adds clarity (see also Nut-12.1).

Dietary indexes or scores are constructed on the basis of a priori hypothesis. Scores are assigned to individuals depending on their adherence to predefined intake amounts, or the population median. The development of the dietary index or score should be described, and whether the aim was to reflect adherence to nutrition recommendations, dietary guidelines, or a certain diet or to predict disease risk. The choice of each index component should be justified, including the cutoff values, because both food and nutrient components could partly reflect similar aspects of the diet, and thus may be highly correlated. Also describe whether there was any weighting of included components and whether variables were energy-adjusted (78, 82).

#### Nut-8.1. Data sources and measurements: describe the dietary assessment method(s) (e.g., portion size estimation, number of days and items recorded, how it was developed and administered, and how quality was ensured); report if and how supplement intake was assessed.

##### Example 1.

“Individual food intake is reported through a semiquantitative FFQ covering the preceding 12-mo period. Between 1992 and 1996, the FFQ included 84 food items, such as edible fats, fruit, vegetables, milk and milk products, bread, potatoes, rice, pasta, fish, meat and meat products, chicken, traditional dishes, hot and cold beverages, sweets, sugar and jam, and snacks. From 1996, this was reduced to 66 food items by deleting entire foods (e.g., liver and kidney) or by merging similar foods (e.g., merging the 2 groups “apples, pears, peaches” and “oranges, mandarines, grapefruit” into one group “apples, pears, peaches, oranges, mandarines, grapefruit”). The 2 data sources have been harmonized and combined into 1 file for the purpose of the food pattern analysis. Portion sizes for the 3 categories of potato/rice/pasta, meat/fish, and vegetables are indicated by participants through comparison with color photos of 4 plates with increasing portion sizes. Frequency of dietary intake is reported on a 9-level scale from none to ≥4 times daily. For the analysis, these frequencies were transformed to a daily frequency” (83).

##### Explanation.

Because each method has different characteristics and utility, clear descriptions of the specific dietary assessment method and the procedure to collect and to analyze dietary data are needed (see Text Boxes 1 and 2). In addition, factors such as the location and time frame of the study (see Nut-5), as well as the mode of collecting dietary data, could potentially influence both the actual diet and the reports of the habitual diet. It is therefore helpful to describe whether the intake information was reported by participants themselves, by participants with assistance from another person, or by proxy. The mode of administration (e.g., face to face interview, telephone interview, questionnaire by mail, Web formula) should also be reported. Furthermore, reporting procedures for quality control, how the quality of collected data were ensured, or both, add clarity. Because dietary assessment is subject to random error and repeated assessments could substantially reduce this error, it is important to clarify whether and how repeated dietary assessments were performed and handled in the dietary analyses, particularly in cohort studies (see Text Boxes 5 and 6).

FFQs typically include a list of food items with questions about how often these are habitually consumed during a given time span (e.g., the previous 12 mo; for details, see Text Box 2). Because there are many varieties of FFQs, each questionnaire needs to be judged for its ability to provide the intended dietary intake information of the specific population. Essential information includes the number of food items and frequency-response categories, as well as how portion sizes were handled. Details of food items should be described, including how they were aggregated and classified, because these are questionnaire- or study specific. If possible, the FFQ should be provided as supplementary material to the article (see Nut-22.2).

Additional details of the FFQ that may be helpful are any control questions included (e.g., number of fish meals consumed per week when the FFQ includes several different items on fish consumption), descriptions of cooking procedures including type of fat used, as well as clear descriptions of questions on dietary supplement use. If the FFQ was intended to capture only certain aspects of the diet (e.g., a short screening questionnaire) or developed for a specific population, this should be clearly stated, and particulars with regard to the validation study should be reported (see also Nut-8.6).

Similar to the FFQ, the dietary history method was originally developed to describe the usual habitual diet of individuals (see Text Box 2). Because the method has had many adaptations and exists in a variety of combinations, it is helpful to describe the methodology and the data collection carefully.

The 24-h recall is a retrospective interview method, aiming to capture the individual’s consumption the preceding day without any previous warning. Any deviation from the original method, such as if the participants were aware of which day of the interview would be carried out or whether the method was a self-instructive Web-questionnaire, should be stated. The number of recall days included and the days of the week (i.e., weekday or weekend) should also be stated (see Text Box 2). How portion sizes were assessed should also be reported. The instructions given to participants before the interview need to be reported, and whether interview aids were provided and if an established interview format was followed.

Food records are collected prospectively, usually by the participants. The number of recorded days (consecutive or not) and the days of the week (i.e., weekday or weekend) should be stated (see also Nut-5). Whether portion sizes were estimated should be reported (e.g., by using photographic aids) or whether foods were weighed or measured (i.e., by using household scales or measurements). It is helpful to include information on the level of detail of the written or oral instructions given (e.g., handling of foods easily forgotten such as water, decomposition of recipes), and if any aids were provided.

Dietary assessment is an area in which considerable methodologic work and development have taken place. Combinations and hybrids of the common assessment methods, and new techniques for recording and reporting (e.g., the Internet and mobile phones), have been developed (44). When new or combinations of procedures and techniques are used, these should be described in sufficient detail and provide further science-based evidence of their specific validity.

#### Nut-8.2. Data sources and measurements: describe and justify food-composition data used; explain the procedure to match food composition with consumption data; describe the use of conversion factors, if applicable.

##### Example 1.

“Total vitamin A was expressed both as retinol equivalents (REs) and as retinol activity equivalent (RAEs) according to the following conversion factors: RE = 1 mg all-*trans* retinol + 1/6 mg dietary all-*trans* β-carotene + 1/12 mg other dietary provitamin A carotenoids; RAE = 1 mg all-*trans* retinol + 1/12 mg dietary all-*trans* β-carotene + 1/24 mg other dietary provitamin A carotenoids. Total vitamin A values were calculated with and without separation of β-carotene isomers in those foods that displayed data for both *trans* and *cis* β-carotene. To calculate vitamin A in REs and RAEs without isomer separation the conversion factor used for all-*trans* β-carotene was adopted for the values of total β-carotene (*trans* plus *cis* β-carotene). Data are shown in the Brazilian Vitamin A Database as micrograms per 100 g edible portion on a fresh-weight basis” (103).

##### Explanation.

In studies of energy, nutrient, and other food component intakes, the food-composition database or other food-composition data need to be described, preferably also giving a reference to the database. Appropriate guidance is needed (e.g., search strategy or references) indicating whether data are directly derived from peer-reviewed publications, monitoring programs, or new analyses. In multicenter studies covering >1 country, the handling of country-specific nutrient values should be described. Factors that influence the quality of the nutrient intake data, such as number of missing values in food-composition data and how these were treated, should be reported. In addition, if applicable, how foods were matched across countries and food databases should be reported. Any conversion factors applied to the consumed food amounts (e.g., raw-to-cooked or precursor-to-bioactive) should be reported, as well as any data handling influencing the food component concentrations (e.g., nutrient retention, yield, or bioactivity).

#### Nut-8.3. Data sources and measurements: describe the nutrient requirements, recommendations, or dietary guidelines and the evaluation approach used to compare intake with the dietary reference values, if applicable.

##### Example 1.

“Estimates of the prevalence of inadequate intakes of essential nutrients from food sources alone were calculated by using the Estimated Average Requirement (EAR) cut-point method. The EARs were primarily derived from the United Kingdom’s Dietary Reference Values. In the case of nutrients for which the EAR was not set (vitamin E, selenium, and iodine), values developed by the Food and Nutrition Board of the Institute of Medicine were used as surrogate EARs. Alternative values were used in addition to the EARs for nutrients for which considerable differences exist in dietary recommendations between countries—that is, folate and calcium—or for which vegetarian-specific recommendations exist—that is, iron and zinc” (104).

##### Explanation.

The recommended approach when reporting the intake adequacy of micronutrients is to evaluate observed intakes against the average requirements (e.g., EAR or Average Requirement) (65). The proportion of the population with intakes below the EAR, or Average Requirement, is the proportion in the study population at risk of inadequate intakes. Only reporting the mean intake in relation to the Recommended Intake or RDA is not sufficient, because this does not enable the reader to judge the adequacy of the diet (65). It is helpful to describe any alternative values used. When the EAR is not available for a specific group and instead calculated (e.g., for children), it is helpful to describe any formulas used.

#### Nut-8.4. Data sources and measurements: when using nutritional biomarkers, additionally use the STROBE-ME; report the type of biomarkers used and usefulness as dietary exposure markers.

##### Example 1.

“Urinary sugars, in particular sucrose and fructose, have been investigated and developed as dietary biomarkers of total sugar intake. If 24-h urine collections are available, sucrose and fructose measured in 24-h urine can be used as predictive biomarkers of total sugar intake. We prospectively investigated the association between sucrose intake and risk of overweight and obesity in a sample of the EPIC (European Investigation into Cancer and Nutrition)-Norfolk cohort study by using urinary sugar biomarkers and self-reported dietary data. Self-reported sucrose intake was significantly positively associated with the biomarker. Associations between the biomarker and BMI were positive (β = 0.25; 95% CI: 0.08, 0.43), while they were inverse when using self-reported dietary data (β = −1.40; 95% CI: −1.81, −0.99). The age- and sex-adjusted OR for BMI (kg/m^2^) >25.0 in participants in the fifth compared with the first quintile was 1.54 (95% CI: 1.12, 2.12; *P*-trend = 0.003) when using the biomarker and 0.56 (95% CI: 0.40, 0.77; *P*-trend < 0.001) with self-reported dietary data. Conclusions: Our results suggest that sucrose measured by objective biomarker but not self-reported sucrose intake is positively associated with BMI” (105)*.*

##### Explanation.

Biological markers of dietary intakes (nutritional biomarkers) are objective measures that are useful in the validation of diet assessment instruments and in studies of diet and disease (see Text Boxes 6 and 7). The use of nutritional biomarkers that reflect dietary exposures will, in combination with self-reported dietary data, strengthen the examination of diet-disease associations (106).

The STROBE-ME provides general guidelines on the reporting in studies that use biomarkers (i.e., not only nutritional biomarkers) (107). Because the type of biological material, sampling method, and choice of analytic method influence the measured concentration of the biomarker, the general guidelines stress the importance of reporting how the samples were collected and handled.

The report needs to indicate if the nutritional biomarker is specific for the dietary exposure, and if it accurately reflects the intake. In addition, it is useful to know if the biomarker is sensitive to an increase in dietary intake (i.e., shows a dose-response association). Readers would also like to know whether the biomarker reflects long- or short-term dietary intake (e.g., through reporting the half-life of the biomarker) and the degree of reliability (reproducibility) of the biomarker (see Text Boxes 6 and 7).

#### Nut-8.5. Data sources and measurements: describe the assessment of nondietary data (e.g., nutritional status and influencing factors) and timing of the assessment of these variables in relation to dietary assessment.

##### Example 1.

“BMI was calculated from weight reported on each biennial questionnaire and height reported at the first questionnaire. Smoking status and number of cigarette use, history of hypertension, aspirin use (number of tablets and frequency of use), regular intake of multivitamins, menopausal status, and use of postmenopausal hormone therapy, parity, and age at first birth were assessed every 2 y” (117).

##### Explanation.

Nondietary data are essential components in studies of diet and health, either as potential confounders or as effect modifiers and intermediate risk factors, of the association between diet and disease. Such nondietary factors are physical (e.g., sex, age, BMI), socioeconomic (e.g., education), genetic, or lifestyle (e.g., physical activity, sedentary behavior, and smoking and alcohol habits) factors. Failure to consider such relevant factors may distort results and lead to incorrect conclusions.

Physical activity represents a particular issue in studies of diet and disease (see Text Box 8). It may be independently associated with outcome, a potential dietary confounder, or both. Estimates of physical activity may also be required when evaluating reports of energy intake as described in Text Box 3.

Physical activity may be estimated by participant self-report with the use of questionnaires or diaries, or by means of objective methods such as pedometers, accelerometers, or heart rate monitors. Many different decisions taken during assessment and data handling will influence the estimated level of physical activity; thus, it is important to report such details. For example, it is helpful to explain how different items in a questionnaire are combined to estimate the PAL, or how estimates of the duration of activities on certain intensity levels were obtained, or how compliance with a recommendation was assessed. Information with regard to the evaluation of the procedure should be included.

Descriptions of how nondietary data were assessed are helpful to enable both understanding of the study and its replication. To facilitate the interpretation of findings, readers need to know the timing of the nondietary data and biomarker collection in relation to the dietary data collection (see also Nut-9). In addition, information on the validity of the methods used should be provided.

Anthropometric measurements (e.g., weight, height, and calculated BMI) are often collected because these measurements are relatively easy to obtain and can be used to evaluate both under- and overnutrition (e.g., obesity is a common risk factor for diet-related chronic diseases). Other simple measures are those related to body fat distribution: for example, waist circumference, waist-to-hip ratio, and skinfold thickness. More advanced measurements of adiposity and body composition can also be of interest. It is important to mention whether these data were obtained through self- or proxy reports or as objective measurements.

When the aim of a study is to identify individuals with nutritional deficiencies, it is essential also to include an assessment of biochemical data, clinical signs of deficiency, or both, because dietary intake assessments alone can only estimate the proportion of a population at risk of nutritional deficiencies (see Nut-8.3).

#### Nut-8.6. Data sources and measurements: report on the validity of the dietary or nutritional assessment methods and any internal or external validation used in the study, if applicable.

##### Example 1.

“We compared FFQ-assessed acrylamide intake with a biomarker of acrylamide intake, hemoglobin adducts of acrylamide and its genotoxic metabolite glycidamide, in a sample of 296 nonsmoking women in the Nurses’ Health Study (NHS) II cohort. The correlation was 0.34 (*P* < 0.0001), adjusted for age, energy intake, BMI, and alcohol intake, and corrected for random within-person variation in the adduct measurement” (123).

##### Explanation.

The published report from an observational study is improved by including information on measures taken when evaluating the validity of the dietary assessment tool (see Text Box 6). This will inform the readers whether the tool actually measures the intended aspect of the diet. Relevant information includes sufficient details about the specific dietary aspect validated, the reference method used, the measures of validity, the population studied, and the sample size (91). If the reference method is another dietary assessment method (i.e., relative validation), details on, for example, number of days, weighed or estimated records, as well as the season and time frame of data collection are useful.

Because no single measure covers all aspects of validity, it is a clear advantage to report >1 approach when describing the validity of a dietary assessment tool (17, 22, 91). Valuable basic information includes whether there is an overall reporting bias (i.e., under- or overestimation of dietary intake), whether there is a dose-response relation (i.e., from partial or single correlation or linear regression analyses) between the estimated intake and the intake measured with the reference method, and whether the validity of a method differs between subgroups.

The understanding of measurement errors in dietary assessment is increasing (97, 102), and techniques have been developed to take measurement error into account when assessing diet-disease associations. Understanding these techniques has resulted in additional emphasis on detailed reporting on the procedures assessing the validity of dietary assessment methods.

#### Nut-9. Bias: report how bias in dietary or nutritional assessment was addressed (e.g., misreporting, changes in habits as a result of being measured, data imputation from other sources).

##### Example 1.

“Diagnoses within 6 mo of food diary completion were excluded to ensure that latent disease without formal diagnosis was not present; otherwise, disease suspected by participants could have influenced their dietary habits. In sensitivity analyses, women with extreme intakes, defined as >1.5 times the IQR >75th percentile, were excluded in tests for linear trends. To investigate the robustness of results to missing data, analyses were repeated by using multiple imputation by chained equations, with imputations based on exposure, covariates, and outcome” (124)*.*

##### Explanation.

Information bias and selection bias are concerns in nutrition research (see Text Boxes 3 and 5), and measures taken to identify or reduce the potential of these biases during all stages of the study (i.e., planning, data collection, data handling, and statistical analysis) need to be reported. When study participants have made changes in their diets (e.g., due to their own or a relative’s disease diagnosis), the reported diet may reflect their present diet correctly. However, such reports may be misleading when examining dietary intakes in relation to health and disease, because the development of chronic disease commonly proceeds over many years.

Some population groups may be at particular risk of misreporting their energy intake (e.g., weight-conscious persons, those who eat out frequently), whereas others (e.g., children) may not be able to report their dietary habits. It will help readers to interpret study findings if information is included about the study setting (see Nut-5), handling of misreporting, and use of any imputations (see Nut-6, -13, and -17; see also Text Boxes 3 and 5).

Information about sampling and self-selection of participants will make it possible for the reader to evaluate the effect of selection as well as the ability to generalize the study findings to the source (or other) populations. Thus, authors ought to describe how subjects were selected, report the characteristics of nonrespondents and dropouts, and discuss how differences might affect observed associations.

Studies may consider the exclusion of participants with potentially biased dietary reports. However, an examination of the robustness of study findings is encouraged, with a subsequent discussion of potential differences between subgroups.

#### Nut-11. Quantitative variables: explain categorization of dietary and nutritional data (e.g., use of N-tiles and handling of nonconsumers) and the choice of reference category, if applicable.

##### Example 1.

“We combined FFQ items to create variables reflecting intakes of *1*) total sugary beverages (combining sugar-sweetened soft drinks, fruit juice, and fruit drinks), *2*) sugar-sweetened soft drinks (high-sugar carbonated beverages, such as cola), and *3*) artificially sweetened soft drinks (sugar-free carbonated beverages, such as diet cola). We created new intake categories to ensure that an adequate number of participants were retained in each intake group across each variable. Cut points were determined before conducting the main analyses based on the relative distribution of intake for each variable. Total sugary beverage consumption was examined as <1/d (reference), 1–2/d, and >2/d; sugar-sweetened soft drink intake was examined as 0/wk (reference), ≤3/wk, and >3/wk; and artificially sweetened soft drink intake was examined as 0/wk (reference), ≤6/wk, and ≥1/d” (125)*.*

##### Explanation.

In nutritional epidemiology, nutrient and food variables are often examined in categories delineated by N-tiles (e.g., quintiles cutoffs indicating fifths of the distribution; see also Nut-14). This is one way of handling outliers, exaggerated intakes (i.e., potential measurement errors), and nonconsumption. Nonconsumption is common in certain foods (e.g., meat) and in alcohol.

The design features of dietary assessment tools may result in exaggerated reports of high intakes. For instance, if many different types of a food item (e.g., fish) are listed in an FFQ this may result in a misleadingly inflated intake in absolute terms (see also Nut-8.1). The true intakes of those individuals who report very high intakes may, however, correctly belong to the higher end of the distribution. In addition, foods with a high concentration of certain nutrients (e.g., vitamin A) may be consumed episodically and unequally in the population, potentially resulting in skewed distributions. Categorization of exposure variables is also needed when a specific cutoff has been recognized, and intakes below or above certain levels need to be compared (e.g., to express the compliance with dietary recommendations). A clear description of the selected categories and cutoffs, the mean or median values of categories, the reference category, and how nonconsumers were handled will be helpful to readers.

In studies that estimate disease risks, the preferred reference category should be one that is stable and includes a sufficient number of study subjects. Although the reference category is often the category with the lowest (or highest) nutrient intake, there may be particular reasons for selecting another category. For instance, individuals who report zero consumption of alcohol may be a mix of those who have never tasted alcohol and those who previously consumed large amounts and recently stopped. In such cases, a more suitable reference may be regular consumers of low amounts. Similarly, a midcategory of the intake distribution might be chosen as the reference category when both high and low intakes are proposed to be associated with the outcome (i.e., U-shaped association).

Excluding nonconsumers in analysis could be informative both in descriptive and etiologic studies but could also bias the findings. In association studies, nonconsumers may serve as reference category for RR estimates, or be the measure of interest. That is, nonconsumers may be maintained in the sample for population mean estimates, or excluded when the average portion size is estimated. If nonconsumers are excluded, their key characteristics should be reported and compared with those of the examined study sample to ensure clarity when interpreting study findings (see Text Box 3).

#### Nut-12.1. Statistical methods: describe any statistical method used to combine dietary or nutritional data, if applicable.

##### Example.

“To best represent long-term diet, we used cumulative average acrylamide intake as our main exposure measure. That is, 1980 intake was used for follow-up from 1980 to 1984; the average of 1980 and 1984 intake was used for follow-up from 1984 to 1986; the average of 1980, 1984, and 1986 was used for follow-up from 1986 to 1990; and so on. This exposure measure also reduces random within-person measurement error over time. In secondary analyses, we used baseline (1980) acrylamide intake only. In addition, we did a latency analysis for breast cancer because of the large number of cases. We used our repeated measures of acrylamide intake to analyze the effect of latency time (time from exposure to cancer) by relating each measure of acrylamide intake to breast cancer incidence during specific periods of latency time: 0–4, 4–8, 8–12, and 12–16 y” (123).

##### Explanation.

A clear description of the statistical methods ensures transparency and enables other researchers to reproduce the study in studies of similar design. Assumptions made when combining data should be stated (see Text Box 9). Studies occasionally combine 2 dietary data collection methods (e.g., FFQ and 24-h recalls). If so, and in order to allow an appropriate interpretation of results, the report should include the method used to combine the dietary or nutritional data and identify the strengths and weaknesses in this approach. When appropriate, a justification for the chosen method is informative.

If dietary patterns are used to represent whole diets, the theoretical basis for the methods should be justified, and any subjective elements in the method clearly identified (see also Nut-7.2). Where possible, the patterns should be fully characterized and the basis presented for any subjective labels (e.g., correlation or covariance matrices, or factor loadings).

The units used should be clearly presented for all variables (e.g., servings per day, portions, grams, millimoles per liter). The same level of detail is equally important for any covariate considered, and the precision of any numbers given should be considered (131). A detailed description of the time frame for dietary intake will help the reader appreciate the appropriateness of the data collection methods and of any modeling assumptions. Similarly, when differential absorption of food and supplemental sources is present, additional care should be taken to describe the methods and models and to state the assumptions made.

#### Nut-12.2. Statistical methods: describe and justify the method for energy adjustments, intake modeling, and use of weighting factors, if applicable.

##### Example 1.

“After examining the distribution of the data, all nutrient intake and biomarker variables were log-transformed to improve normality. We used the residual method to adjust dietary FAs and carotenoids for total energy by regressing nutrient intakes on *1*) self-reported total energy intake derived from FFQs and *2*) body weight and physical activity” (132).

##### Example 2.

“The macronutrient intake is reported as absolute intake (grams per day) and as a percentage of energy, except for fiber, which is presented as grams per day and grams per mega-Joule. Micronutrient intake is presented as nutrient density (i.e., the amount of reported intake per 4.2 MJ); Nordic nutrition recommendations were used as a reference. For micronutrients, the recommendations were converted to nutrient density by dividing the recommended nutrient intake with the recommended energy intake multiplied by 4.2 MJ” (133).

##### Explanation.

Individuals with high energy intakes might have a higher consumption of many food components. Therefore, failure to adjust nutrient intakes for energy intake could lead to misleading conclusions with regard to the link between dietary intakes and disease (see Text Box 4). In addition, energy adjustment will potentially remove some of the negative influence of dietary measurement errors (80, 81). The method used for energy adjustments (i.e., residual or nutrient density) should be described.

It is also recommended to describe whether the energy adjustments include or exclude energy from any particular food or nutrient. For example, in studies in which alcohol is a strong risk factor for the disease (e.g., in studies on breast cancer), and there is a need to examine alcohol use separately, nonalcohol energy may be used instead of total energy when computing nutrient densities or nutrient residuals to examine dietary exposures. It is helpful to describe the statistical techniques used to remove the within-person error when using short-term instruments, such as 24-h dietary recalls, to estimate the proportion of a population below or above a recommendation or cutoff.

#### Nut-12.3. Statistical methods: report any adjustments for measurement error (i.e., from a validity or calibration study).

##### Example.

“A second FFQ was taken from a sample of 1918 (5%) of the cohort, from which the amount of random measurement error was estimated by using a regression calibration approach to obtain individual predicted values of dietary exposure for all participants. Cox proportional hazards regression was then conducted by using the predicted values for each individual categorized into quintiles to give estimated HRs corrected for some of the effects of measurement error. 95% CIs were obtained from bootstrapped estimates” (39).

##### Explanation.

Despite the improvement in dietary assessment methods, random and systematic measurement errors, both within and between individuals, may be present in dietary data. The statistical understanding of dietary measurement errors is increasing (97, 102), and different methods have been developed to try to correct for measurement errors in analysis when examining associations between dietary exposures and disease risks (17, 134). Because these methods are all based on specific assumptions, and depend on the type of calibration study and data available, there is a need to clearly describe them in order to improve the interpretation. It is helpful to provide the rationale for the adjustment as well as to describe the adjustment method, including risk estimates with 95% CIs (see also Text Box 5).

### Results

#### Nut-13. Participants: report the number of individuals excluded on the basis of missing, incomplete, or implausible dietary and nutritional data.

##### Example.

“We excluded participants with cancer, implausible energy intakes (reported as <600 or >3600 kcal/d for women and <800 or >4200 kcal/d for men; 1 kcal = 4.18 kJ), or missing alcohol intake at baseline” (135).

##### Explanation.

Missing and implausible data are omnipresent in dietary assessments and may introduce bias or attenuate associations (see also Nut-9 and -17 and Text Boxes 3 and 5). Individuals with biologically extreme values are commonly excluded. To enable the reader to better evaluate the study, information with regard to the final study power and any bias is needed. It is helpful to describe the number and characteristics of excluded individuals due to missing or incomplete dietary data. Also describe any sensitivity analyses performed to explore the robustness of study findings.

#### Nut-14. Descriptive data: give the distribution of participant characteristics across the exposure variables, if applicable; specify if food consumption for the total population or consumers only was used to obtain results.

##### Example.

**FIGURE 1:**
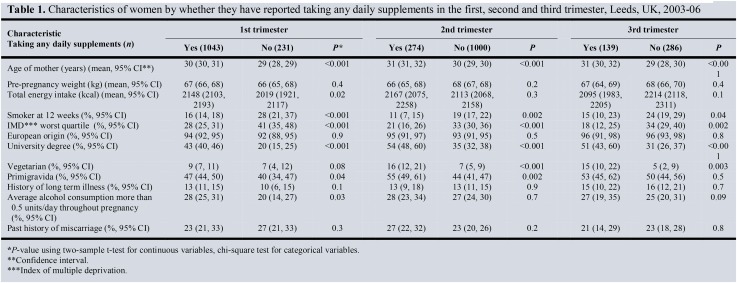
Example table. Reproduced from reference 136 with permission.

An example table is shown in **Figure 1**.

##### Explanation.

Confounding is a major concern in nutritional epidemiology, because most dietary exposures are interdependent, and many socioeconomic and lifestyle factors covary with dietary exposures (see also Nut-8.5). Reporting participant characteristics across the dietary exposure variables will enable the reader to assess the potential impact of confounders.

#### Nut-16. Main results: specify if nutrient intakes are reported with or without the inclusion of dietary supplement intake, if applicable.

##### Example.

“There was no overall association between intake of vitamin C and the risk of developing hypertension. Comparing individuals in NHS-I whose daily consumption of vitamin C was ≥1500 mg with those whose intake was <250 mg in the other 2 cohorts, the RRs (95% CIs) were 1.02 (0.91, 1.14) in NHS-II and 1.06 (0.97, 1.15) in the Health Professionals Follow-Up Study. In a secondary analysis, we excluded women and men who took supplemental vitamin C (including multivitamin users) and analyzed the association between dietary intake of vitamin C and incident hypertension. Comparing individuals whose daily dietary consumption of vitamin C was ≥250 mg with those who consumed <100 mg/d, the adjusted RRs (95% CIs) were 1.05 (0.97, 1.14) in NHS-I, 1.06 (0.92, 1.23) in NHS-II, and 0.99 (0.84, 1.17) in the Health Professionals Follow-Up Study” (137).

##### Explanation.

The total intake of nutrients could be underestimated if supplement use is not accounted for. It may be helpful to the reader if nutrient intakes are presented both including and excluding the contribution from supplements (see also Nut-8.1). However, depending on the study aim and the data available, it could be more suitable to present supplement use as a separate exposure, or as a covariate. Because both the chemical form and the dose of nutrients found in supplements often differ compared with nutrients found in foods, dietary supplements may have a different effect than food-derived nutrient exposure. In addition, when only less-detailed dietary supplement data are available (e.g., current, ever, or never use), it may not be possible to combine dietary and supplement data. Where differential absorption of food and supplemental sources is relevant, additional care should be taken to describe the methods of data collection and analysis. Any assumptions made should also be stated.

#### Nut-17. Other analyses: report any sensitivity analysis (e.g., exclusion of misreporters or outliers) and data imputation, if applicable.

##### Example 1.

“Individuals with dietary change in the past are suspected to have unstable food habits. Dietary change in the past (yes or no) was derived from the questionnaire item: “Have you substantially changed your eating habits because of illness or for some other reason? All analyses were performed in *1*) all individuals, *2*) individuals reporting adequate energy intake (i.e., nonadequate reporters were excluded), and *3*) individuals reporting stable dietary habits (i.e., individuals reporting dietary change were excluded)” (138).

##### Explanation.

Misreporting of dietary intake is common and a major challenge to nutritional epidemiology, especially underreporting, which is likely related to personal characteristics and may be associated with health outcomes (see Text Box 3). Depending on the study design and available data, researchers may select different approaches to examine the robustness of study findings and thus enhance the understanding of the impact of measurement errors. Individuals may have changed their diets before the start of the study due to ill health (e.g., diagnosed with diabetes or hyperlipidemia) or other reasons. In such cases, the reported diet may not be relevant for the outcome assessed, and therefore it may be sensible to repeat analysis excluding subgroups of the study sample (see Text Box 5).

It is often helpful to compare the reported energy intake with the TEE calculated from estimates of the resting energy expenditure and the PAL (see Text Boxes 3 and 8). This will enable readers to evaluate if under- or overestimation of dietary energy is present. Although studies often exclude individuals with high or low reported energy intakes, this may not always be appropriate due to excluding some true intakes. Alternative solutions could include a separate assessment of these groups (see Text Box 3). If individuals with extreme values (i.e., clearly not compatible with biological function) are excluded, the allowable range for those included should be stated.

Another concern is missing values in FFQs, especially when dietary information is combined in nutrient intake calculations or in indexes. Some missing values in an FFQ may represent random mistakes, whereas others reflect nonconsumption (see Text Box 3). To understand the procedure and enable replication of the study, details of any imputation and the statistical handling need to be provided.

### Discussion

#### Nut-19. Limitations: describe the main limitations of the data sources and assessment methods used and implications for the interpretation of the findings.

##### Example.

“However, the dietary history method used has limitations that may have caused some misclassification of subjects. These tend to diminish the associations observed between exposure and outcome. The result of the dietary history interview is always a subjective assessment of the respondent’s own dietary habits. A period of 1 y is a lengthy time to recall. Food models were used to diminish errors in recall, and open-ended questions enabled respondents to be more specific in their answers. To minimize possible bias, trained nutrition professionals used a structured questionnaire. In general, the short-term repeatability of the dietary history method was relatively good. However, rather poor repeatability for glucose and fructose hinders the interpretation of the results and the possibility of chance findings increases. The poorer long-term consistency can be partly explained by changes in Finnish dietary habits. Changes in food consumption during follow-up tend to weaken the associations observed. For this reason, follow-up in this study was limited to 12 y” (139).

##### Explanation.

Given the complexity of nutritional epidemiology, the discussion of study limitations is an essential part of the scientific reporting. Assumptions with regard to the accuracy of the reported dietary intake should be handled with care (see also Text Box 6). Potential sources of biases and, if relevant, how these were handled, as well as degrees of error related to the dietary assessment need to be reported and thoroughly discussed when interpreting the results. To observe different health outcomes in exposed compared with nonexposed study participants, the dietary exposure gradient needs to be large enough.

#### Nut-20. Interpretation: report the nutritional relevance of the findings, given the complexity of diet or nutrition as an exposure.

##### Example 1.

“A dilemma in the present study is the difference in group size. In order to avoid misinterpretations of the results and in order to deepen our understanding when analyzing data, estimations of effect sizes were calculated. Having a large sample increases the risk of overvaluing observed significant differences where the importance of the differences could be quite trivial. This occurred, for example, when we compared the differences of reported intake between the 2 nonceliac referent groups (data not shown) and found many significant differences; however, the estimated effect size revealed that the relevance of these differences was mostly small. On the other hand, a calculated large effect size on nonsignificant differences in a small sample, such as the changes in the previously diagnosed celiac disease group between baseline and follow-up, suggests a need for further research with a larger sample size” (140).

##### Explanation.

The nutritional relevance of the findings depends on a number of factors. The quality of the dietary data will determine the ability to detect significant associations. Small dietary differences without any biological significance could in large cohort studies result in significant associations with disease outcomes. Reporting an effect size of intake differences (141) may facilitate the understanding of the practical and theoretical utility of study results. Translating an increased risk into a reduction in survival in number of months may also make it easier to judge the relevance of findings. The inherent complexities of diet as an environmental exposure pose additional challenges to the interpretation of study findings, which requires careful consideration and nuanced and balanced conclusions.

Nutrients and other bioactive substances are generally not consumed in isolation. Food contains various bioactive substances, and each meal typically consists of a combination of several foods. It might be difficult to distinguish the “true” effect of a single nutrient, because nutrients interact with each other, with other compounds, and with the surrounding food matrix in complex ways (142). When intercorrelated nutrients (e.g., different FAs) are examined together, there is a risk of attenuated associations; however, on the other hand, if not analyzed together, the separate effects of intercorrelated nutrients may be impossible to detect. The dietary concentration of a single nutrient may also be too low to detect any health effect (77). Moreover, dietary habits cluster with other health behaviors. Lifestyle factors other than diet and environmental factors, as well as the physiologic and disease status of study participants, will also influence the impact of dietary exposures. Indicate whether conclusions were based on analyses of dietary intakes alone or whether intakes through diet were combined with dietary supplements (see Nut-16).

The variation in food habits across populations, and across subgroups within populations, further complicates the interpretation and contributes to inconsistencies between studies. For example, meat and meat products are major sources of saturated fat in the United States, whereas dairy products dominate in the Nordic countries, resulting in diverging dietary covariates and potential confounders (i.e., dietary components related to fat intake will vary). Similarly, dietary carbohydrates are largely contributed by fruit and vegetables in Southern European countries, whereas sugary foods, cereals, and potatoes are major contributors in Northern Europe (143, 144). Thus, the food habits in the population under study should be considered when discussing the generalizability of results, and the consistency of diet-disease associations needs to be examined in different populations.

### Complementary material

#### Nut-22.1. Ethics: describe the procedure for consent and study approval from ethics committee(s).

##### Example 1.

“Before data collection, written consent was obtained from parent participants in the original data collection for the Early Childhood Longitudinal Programs, Birth Cohort (ECLS-B). The National Center for Education Statistics approved our use of the deidentified and anonymized restricted-use data set for the current analysis. The Johns Hopkins Institutional Review Board deemed that this analysis of deidentified secondary data involved non–human subjects research” (145).

##### Explanation.

As stated in the Helsinki Declaration (146), ethics apply to all types of medical research concerning human subjects that includes research on identifiable human material or data. The Council for International Organizations of Medical Sciences has recently published a new version of its International Ethical Guidelines for Health-Related Research Involving Humans (147). It is useful to provide details about ethical approval, if it has been granted, and by whom. The need for ethical approval for observational studies, however, varies across countries (148) (see also Nut-22.2).

Regardless of the legislation available in the country of research, all research studies collecting data from human participants impose ethical obligations to participants (149). Therefore, researchers should ensure clarity and describe how they addressed the ethical issues in their research, including the research risks of harm. In addition, the procedures to guarantee data privacy and confidentiality during the analysis and handling of personal data should be clearly described (150).

#### Nut-22.2. Online material: provide data collection tools and data as online material or explain how they can be accessed or why they cannot be provided.

##### Example 1.

“Dietary information was collected by using a 121-item, self-administered FFQ. Details of which foods were included in each food group are listed in the online Appendix” (151).

##### Explanation.

Traditionally, efforts to share research have focused on manuscripts that provide a narrative summary of the conducted study and the results. However, other research outputs, such as protocols, data collection instruments, software, and algorithms, are essential for the interpretation of findings and the reproduction of the research project. An increasing number of journals allow researchers to upload supplementary online material or to link objects to online sources or repositories. This is an opportunity to maximize the build-up of scholarly knowledge and is increasingly recognized as an integral part of good research practice and academic culture.

There are ongoing discussions about ethical aspects of data-sharing: for instance, within European data infrastructure initiatives [e.g., Biobanking and BioMolecular Resources Research Infrastructure–European Research Infrastructure Consortium (www.bbmri-eric.eu) (152) and the European Clinical Research Infrastructure Network (www.ecrin.org) (153); see also Nut-22.1].

Data-sharing could vary from sharing information with regard to the study design, the mode of data collection and outcomes, the number of participants, and a full list of available measurements, up to the software used and individual, anonymized data. A tangible benefit of sharing protocols and hypotheses for epidemiologic studies in public repositories is that duplication of efforts potentially would be avoided. Legitimate data exploration and discovery could be cost-effective and maximize the impact of available epidemiologic data and should not be limited by preregistration of protocols. Important caveats apply, however, and whether epidemiologic studies should be preregistered or not is debated (154–160).

Researchers are encouraged to provide access to the data needed to reproduce the results. Various research-funding agencies, universities, and scientific journals have adopted policies that allow data to be accessible for the reproduction of the study findings; and different data repositories are being developed for such purposes. To ensure an effective use of data for future research and scientific discovery, data-sharing needs to be organized so that interaction with computational agents is facilitated. In other words, data should be “findable, accessible, interoperable, and reusable” (FAIR) (161).

Research information with regard to humans should be managed with the highest and most appropriate ethical standards (see Nut-22.1). Efforts to ensure that research data are available include collection and storage of high-quality information with long-term validity. In order to do this, data must be well documented, so that other researchers can access, understand, and use these data, and add value to the original data independently of the original investigators. Furthermore, there is a need for high-quality stewardship of scientific data and adequate procedures, including long-term care, quality control, and adequate commitment as well as resources to handle the data. Funding bodies such as the United Kingdom Medical Research Council (162), the NIH (163), and the European Commission (164) now explicitly require statements around data management. All applicants submitting funding proposals to these (and many other) funding agencies are required to include a Data Management Plan as an integral part of their application.

### Concluding remarks

The STROBE-nut is intended to complement the STROBE recommendations (3, 6) and to help improve and standardize the reporting of dietary data in nutritional epidemiology publications. To improve the checklist, feedback and the submission of other examples or good practices are encouraged through our website (www.strobe-nut.org) (8).

In 2008, the international network Enhancing the QUAlity and Transparency Of health Research (EQUATOR) was launched, which aimed to improve reporting and to increase transparency of all types of health research studies. The EQUATOR website (http://www.equator-network.org) (165) is a useful online resource.
